# Supplementary material for: Efficient pure blue hyperfluorescence devices utilizing quadrupolar donor-acceptor-donor type of thermally activated delayed fluorescence sensitizers
Source: Nat Commun. 2023 Jan 26;14:419. doi: 10.1038/s41467-023-35926-1 (PMC9876909; doi:10.1038/s41467-023-35926-1)
Supplement: Supplementary file 1 — Supplementary Information [file 41467_2023_35926_MOESM1_ESM.pdf]

Supplementary Information

Efficient pure blue hyperfluorescence devices utilizing quadrupolar donor-acceptor-donor type of thermally activated delayed fluorescence sensitizers

Hyuna Lee[1, ‡], Ramanaskanda Braveenth[1, ‡], Subramanian Muruganantham[1], Chae Yeon Jeon[1], Hyun Seung Lee[1], and Jang Hyuk Kwon[1,*]

[‡]These authors contributed equally

[1] Organic Optoelectronic Device Lab (OODL), Department of Information Display, Kyung Hee University, 26, Kyungheedae-ro, Dongdaemun-gu, Seoul, 02447, Republic of Korea

* Corresponding author; E-mail: jhkwon@khu.ac.kr

Supplementary Figures

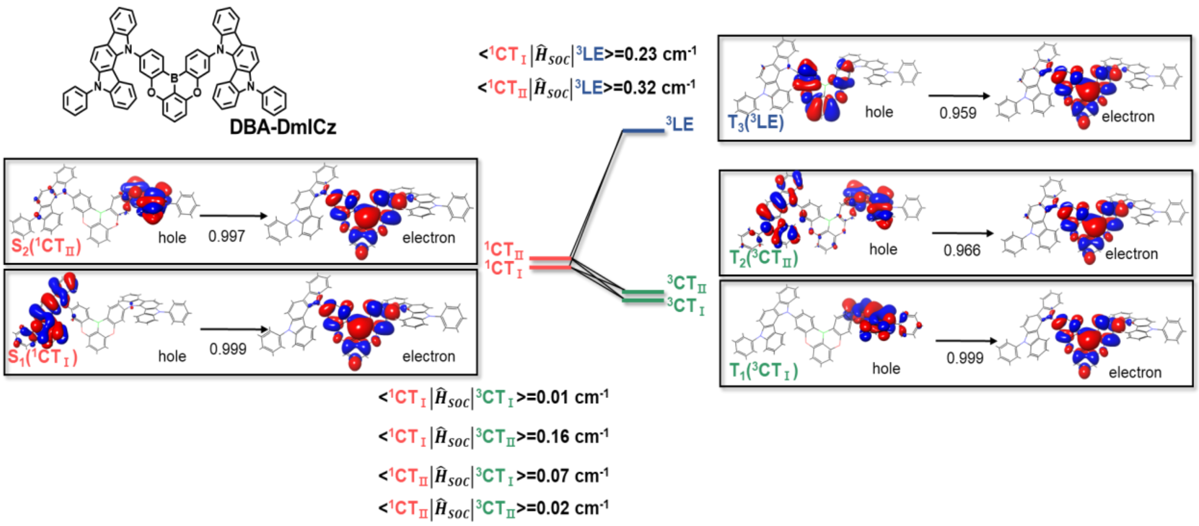

Supplementary Fig. 1 NTO distribution and corresponding SOCME values of DBA-DmICz.

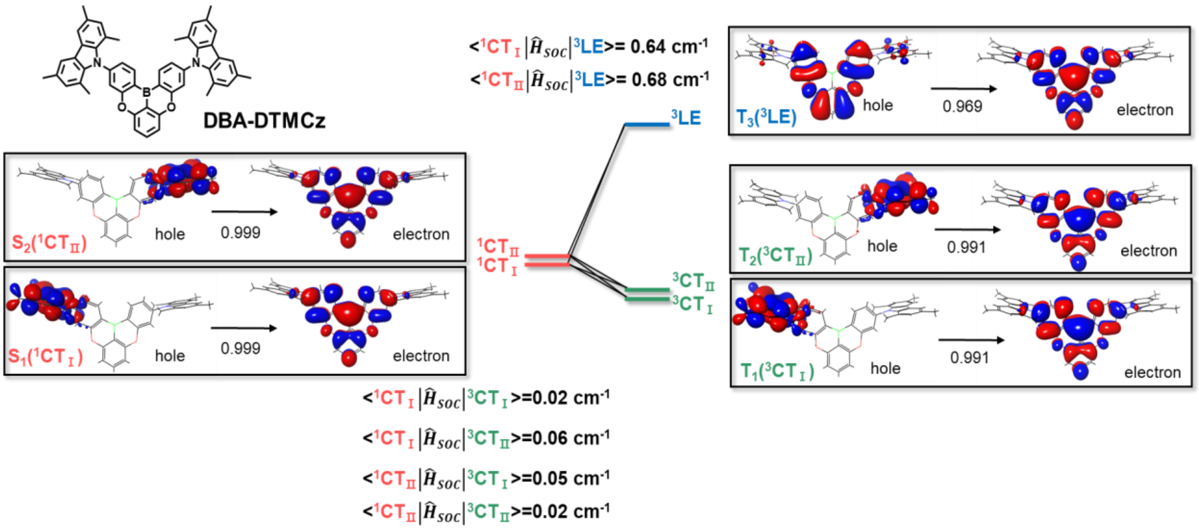

Supplementary Fig. 2 NTO distribution and corresponding SOCME values of DBA-DTMCz.

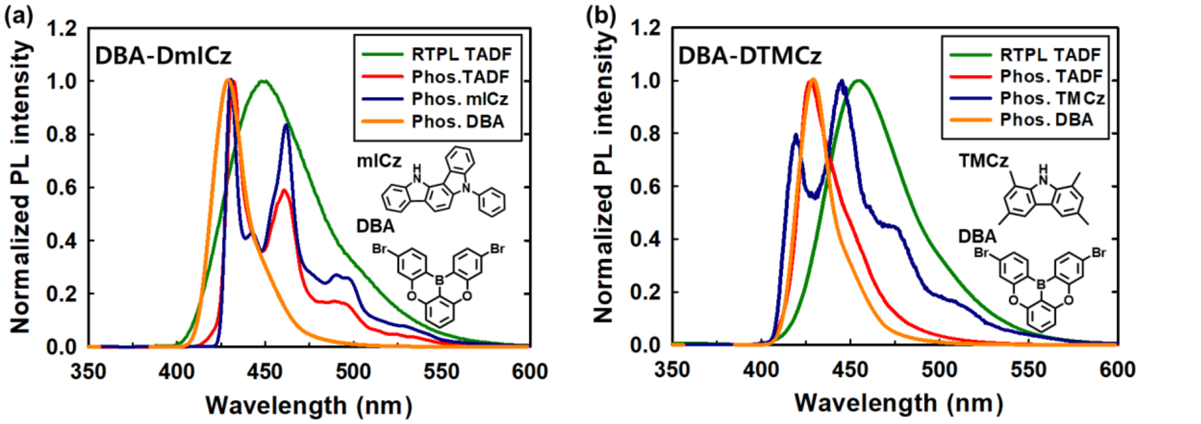

Supplementary Fig. 3 Phosphorescence spectra of TADF, donor, and acceptor moieties of (a) DBA-DmICz and (b) DBA-DTMCz in 10^{-5} M of toluene solution. The RTPL spectra were measured in 300K and the phosphorescence spectra were measured in 77K with 30 ms of delaying.

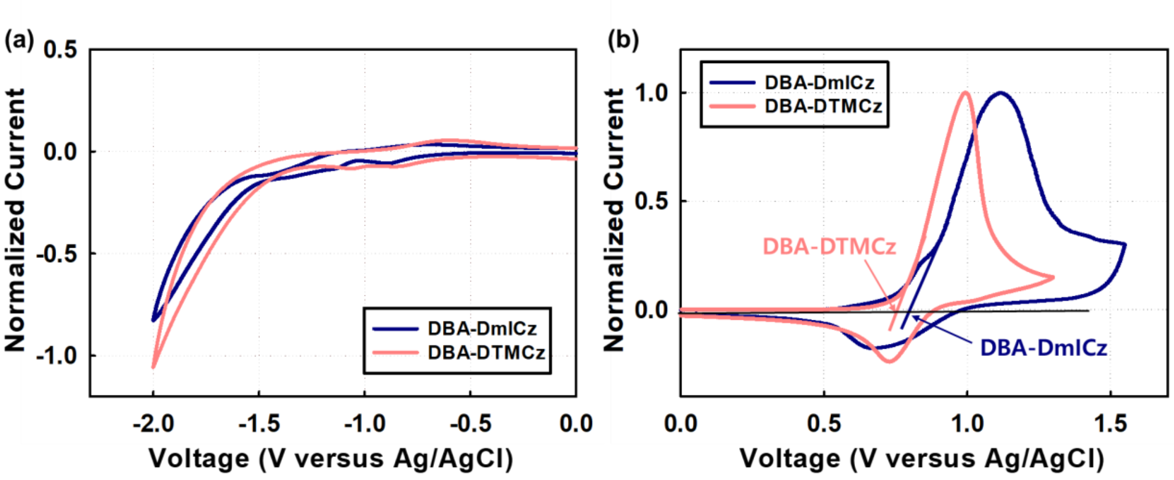

Supplementary Fig. 4 (a) Reduction and (b) oxidation cyclic voltammetry (CV) curves of DBA-DmICz and DBA-DTMCz.

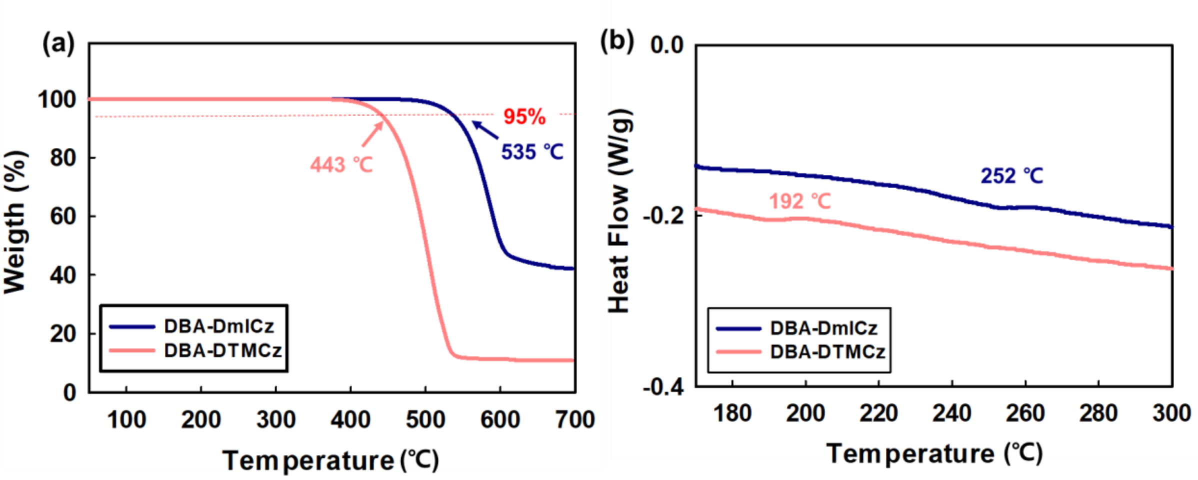

Supplementary Fig. 5 (a) TGA, and (b) DSC graphs of DBA-DmICz and DBA-DTMCz.

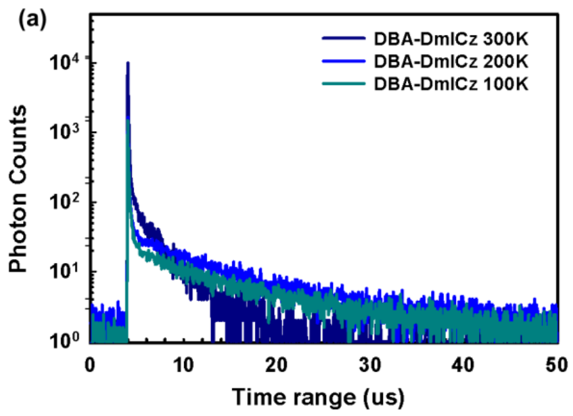

Supplementary Fig. 6 Temperature dependent TRPL measurement of (a) DBA-DmICz and (b) DBA-DTMCz.

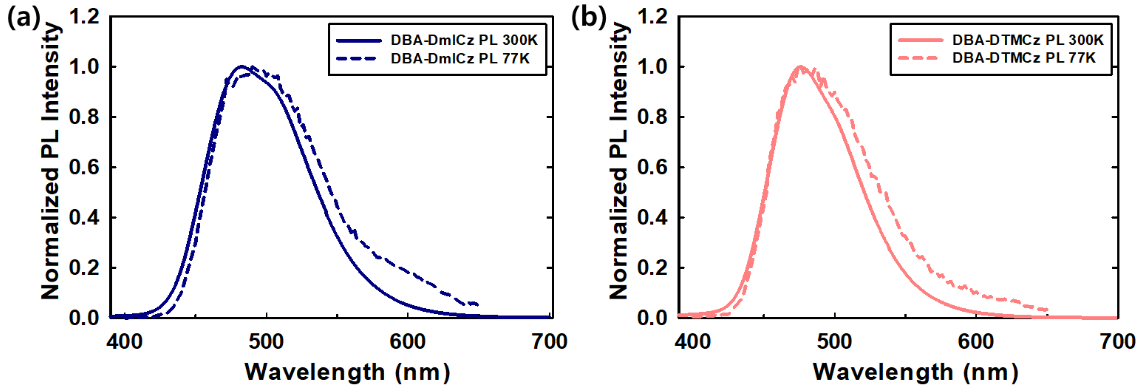

Supplementary Fig. 7 The PL and phosphorescence spectra (77K) in 30% doped film state of (a) DBA-DmICz and (b) DBA-DTMCz.

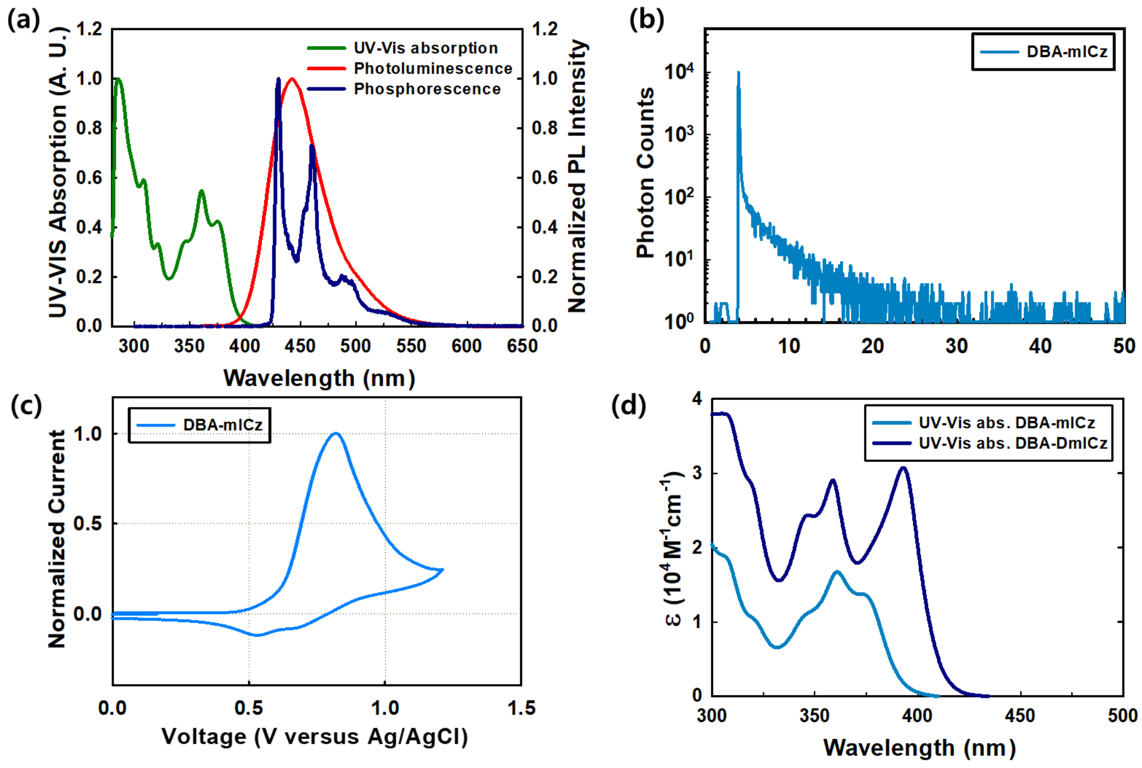

Supplementary Fig. 8 (a) The UV-Vis absorption, PL, and phosphorescence spectra of DBA-mICz in toluene state. (b) TRPL measurement in 30% doped film state and (c) CV measurement of DBA-mICz. (d) The comparison of UV-Vis absorption spectra between DBA-mICz and DBA-DmICz at a concentration of $1{\times}10^{-4}$ M.

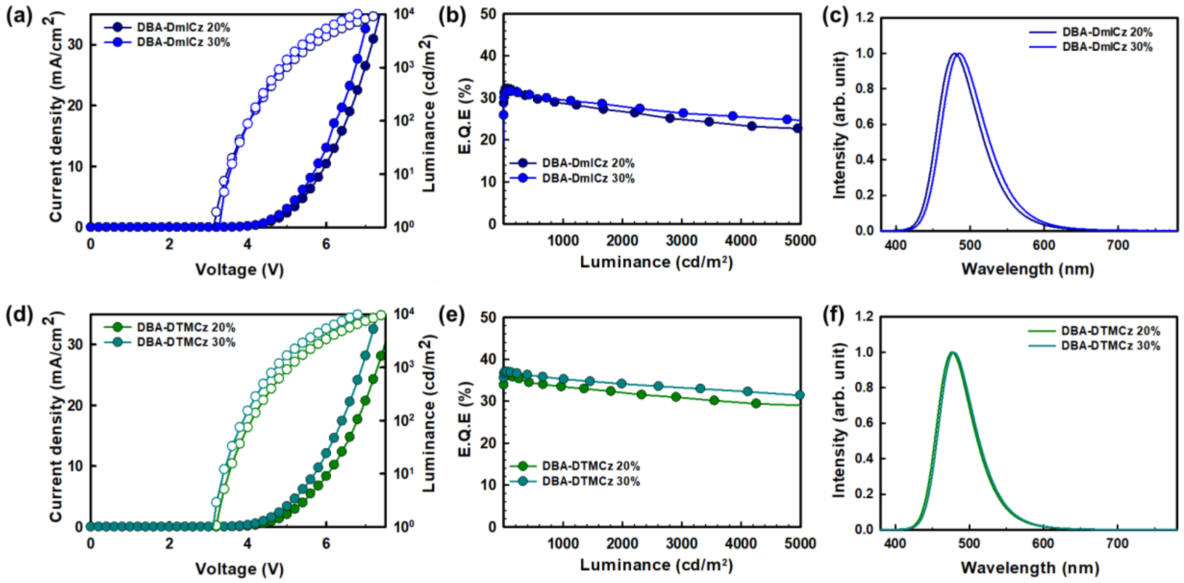

Supplementary Fig. 9 Device performances with different doping concentration of (a) *J-V-L* curves (b) EQE versus luminance (c) The electroluminescence spectra of devices DBA-DmICz (d) *J-V-L* curves (e) EQE versus luminance and (f) The electroluminescence spectra of devices DBA-DTMCz.

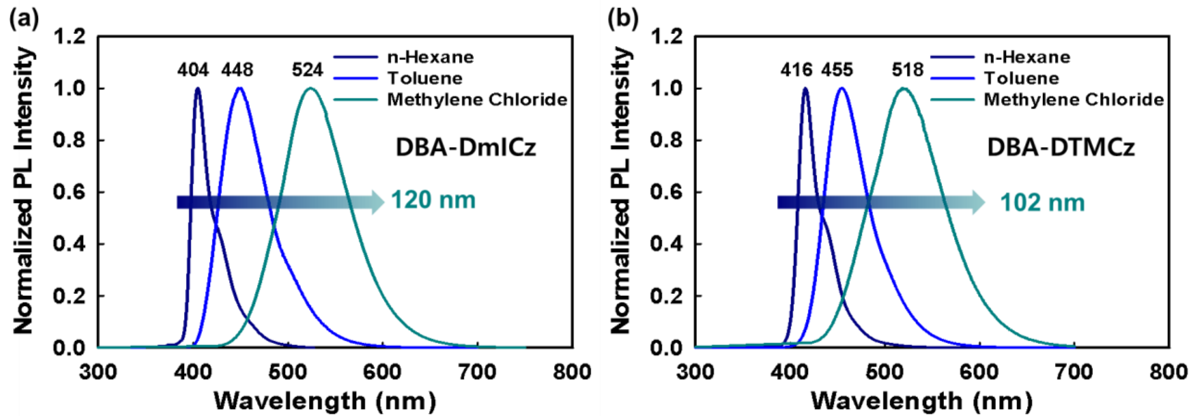

Supplementary Fig. 10 Measurement of Solvatochromism effect from *n*-Hexane to Methylene Chloride. (a) DBA-DmICz and (b) DBA-DTMCz.

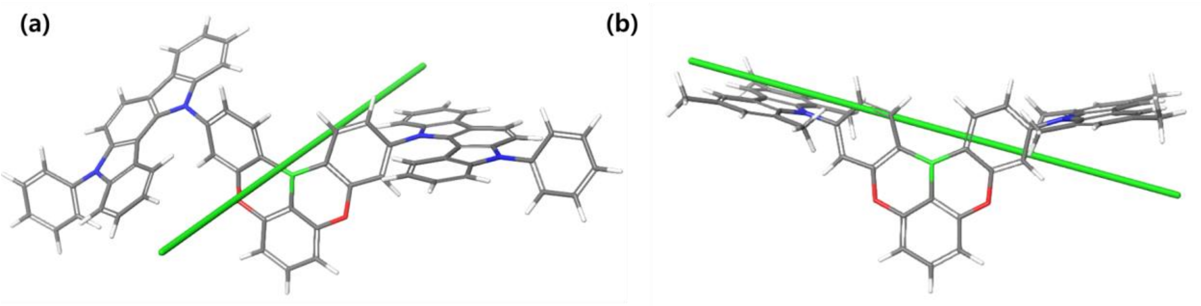

Supplementary Fig. 11 Calculated transition dipole moment of (a) DBA-DmICz and (b) DBA-DTMCz.

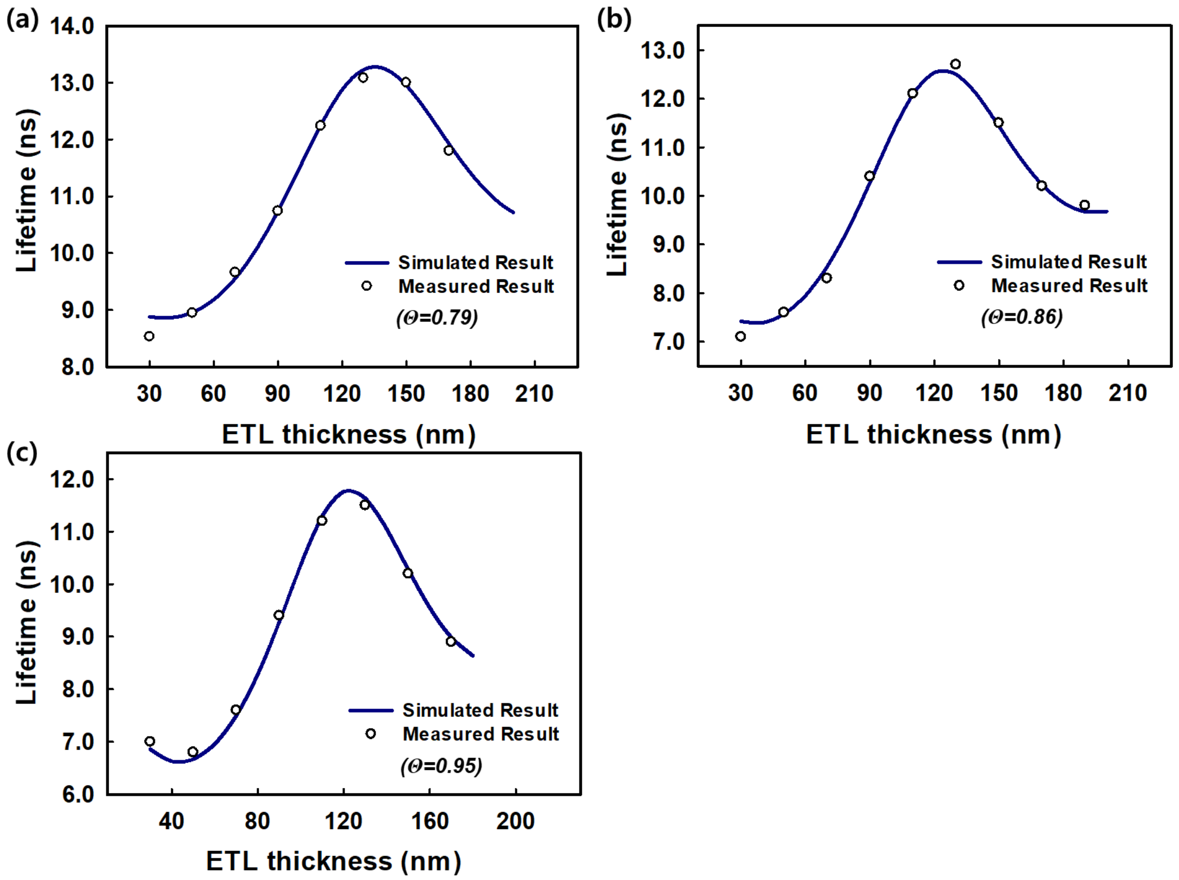

Supplementary Fig. 12 Measurement of horizontal orientation ratio of (a) DBFPO: 30% of DBA-DmICz, (b) DBFPO: 30% of DBA-DTMCz, and (c) DBFPO: 30% of DBA-DTMCz: 1% of *v*-DABNA.

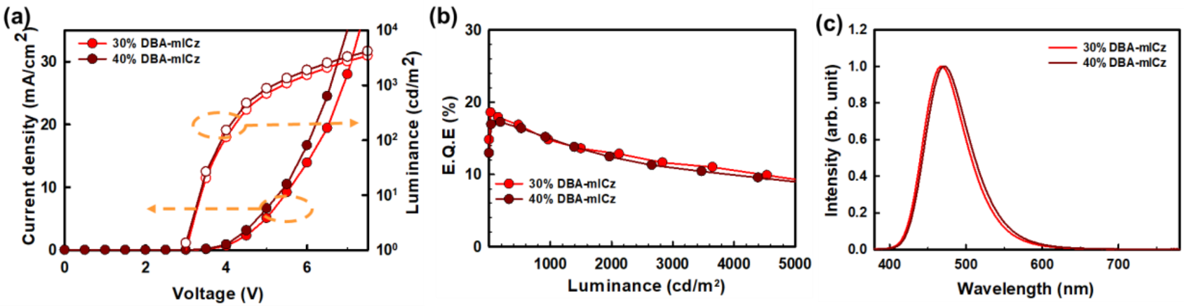

Supplementary Fig. 13 Device performances of DBA-mICz with 30 and 40% of doping concentration (a) *J-V-L* curves (b) EQE versus luminance and (c) The electroluminescence spectra of devices.

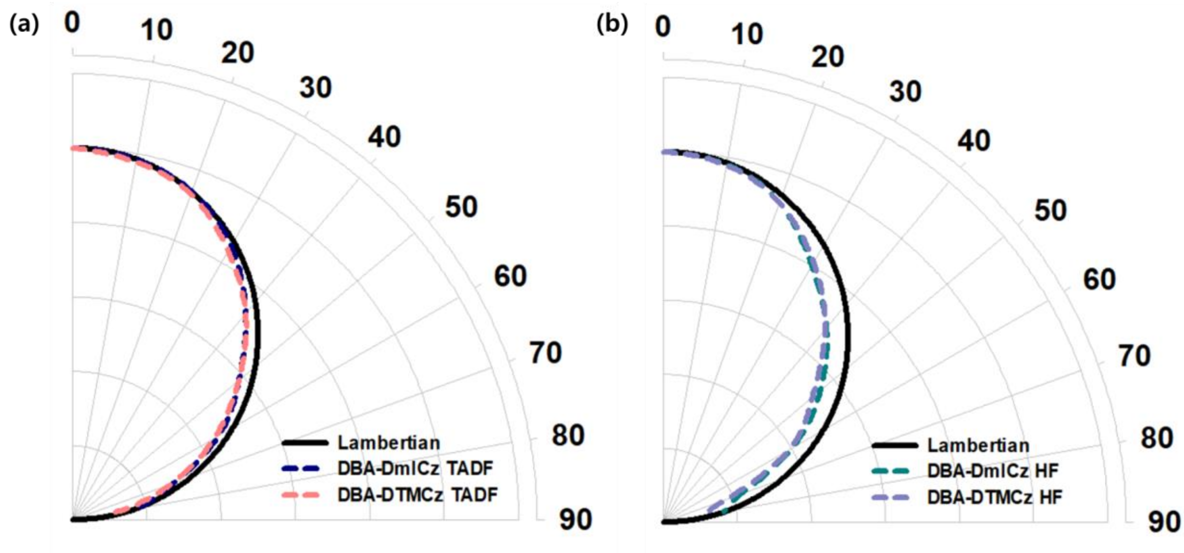

Supplementary Fig. 14 Angular-distribution emission pattern of (a) TADF, and (b) HF device.

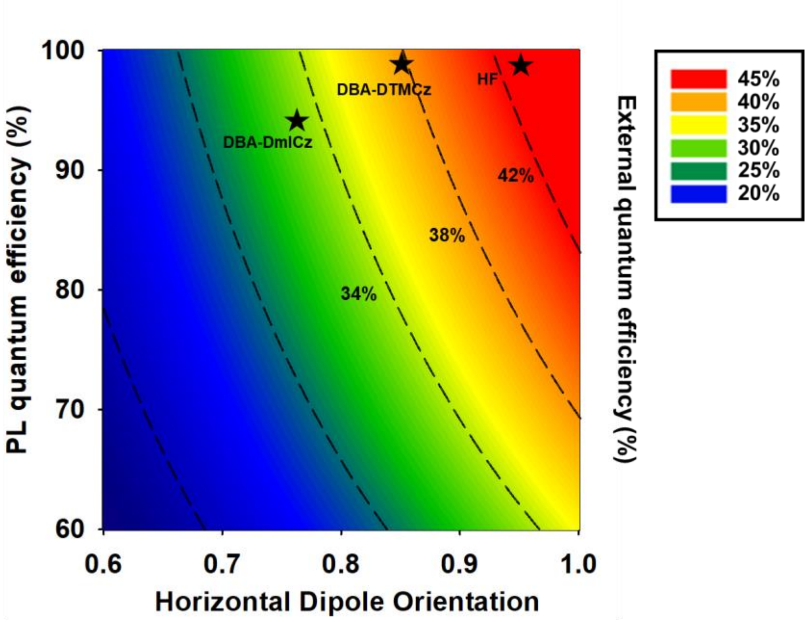

Supplementary Fig. 15 Calculated theoretical EQE values depending on the PLQY and horizontal orientation factor.

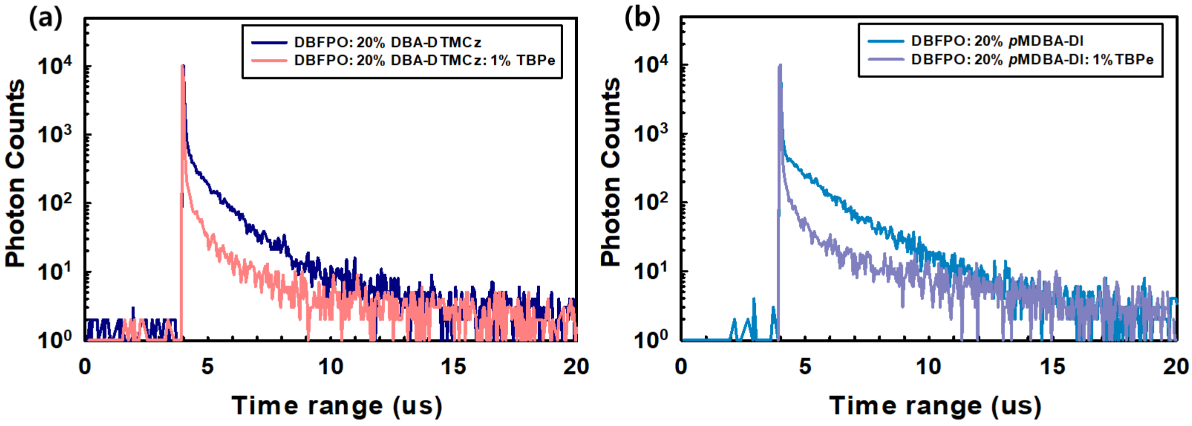

Supplementary Fig. 16 The TRPL measurement of TADF and HF system utilizing (a) DBA-DTMCz and (b) *p*MDBA-DI. The photoexcitation wavelength was 340 nm.

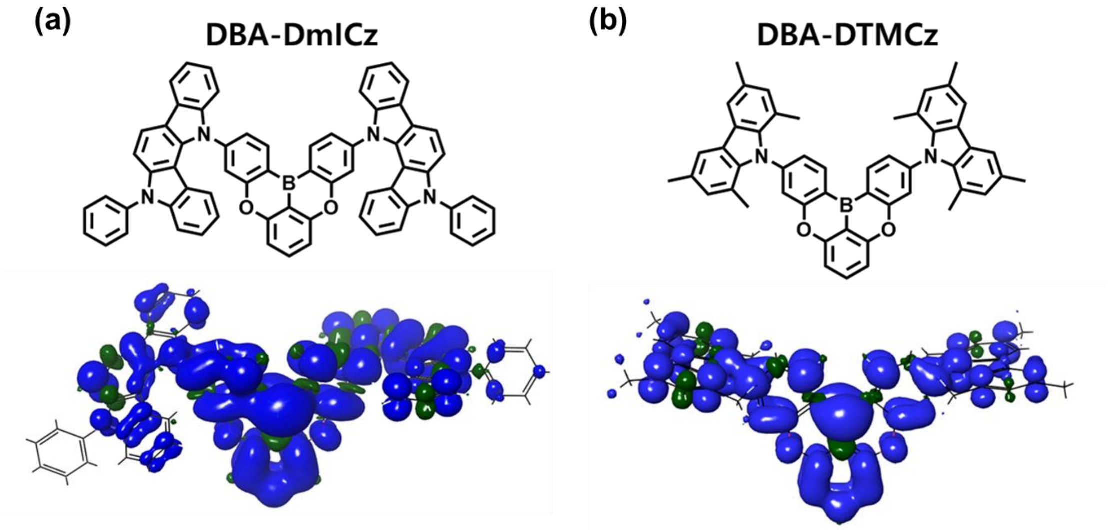

Supplementary Fig. 17 The triplet density distribution of (a) DBA-DmICz, and (b) DBA-DTMCz.

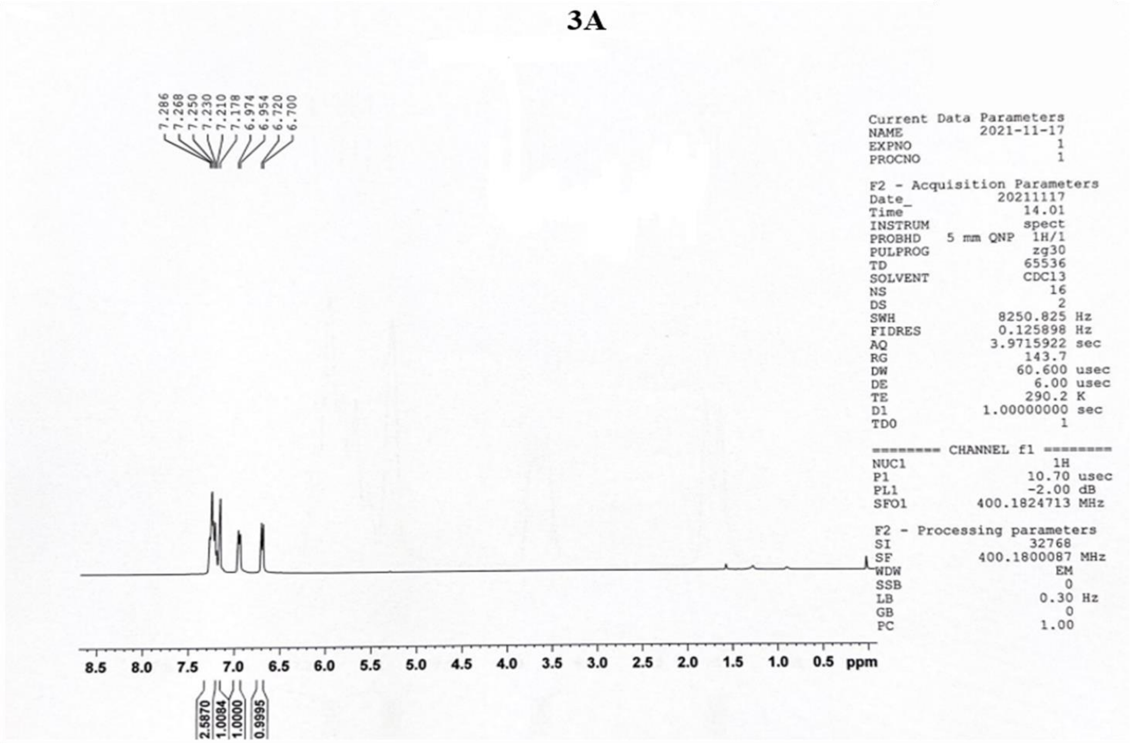

Supplementary Fig. 18 Proton NMR of 3,3'-((2-iodo-1,3-phenylene)bis(oxy))bis bromobenzene) (3A).

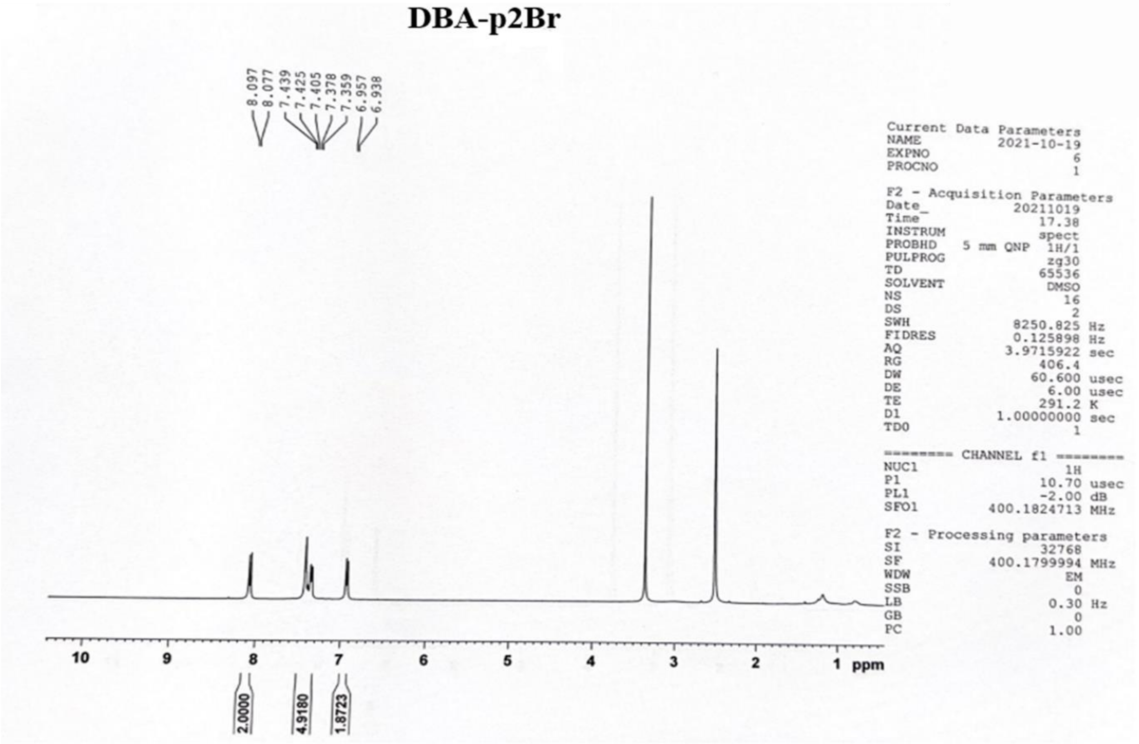

Supplementary Fig. 19 Proton NMR of 3,11-dibromo-5,9-dioxa-13b-boranaphtho[3,2,1-de]anthracene (DBA-p2Br).

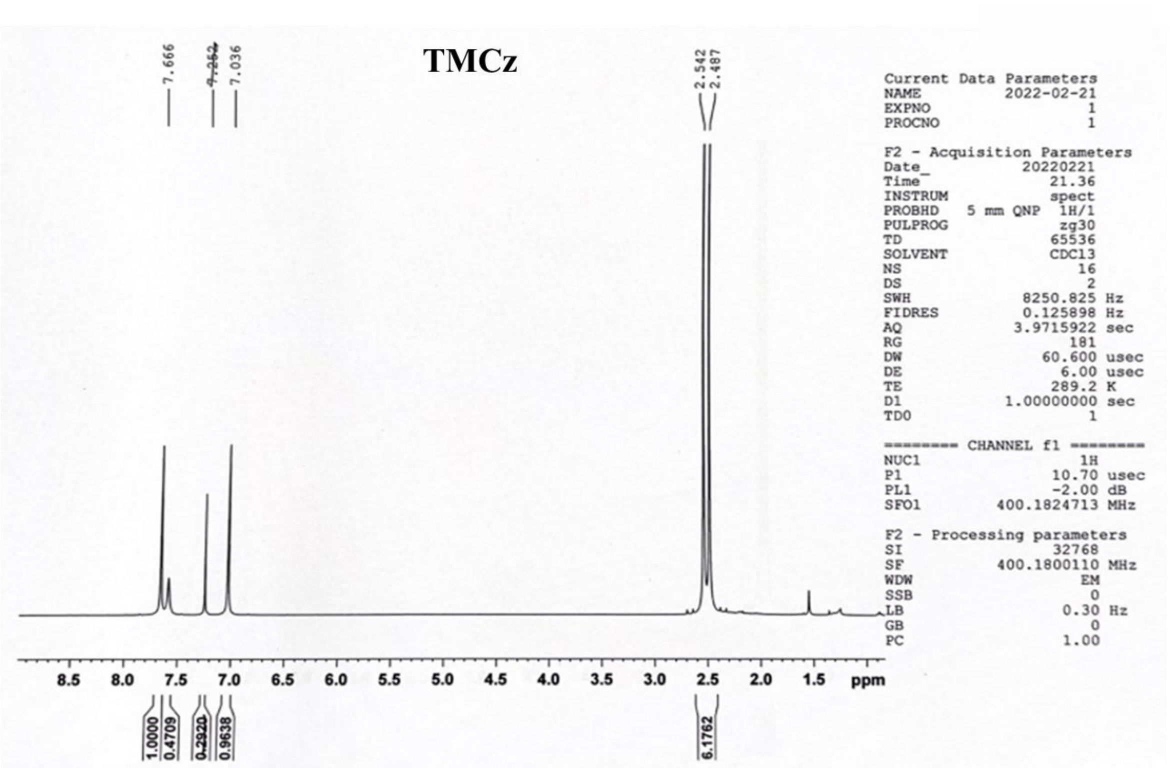

Supplementary Fig. 20 Proton NMR of 1,3,6,8-tetramethyl-9H-carbazole (TMCz).

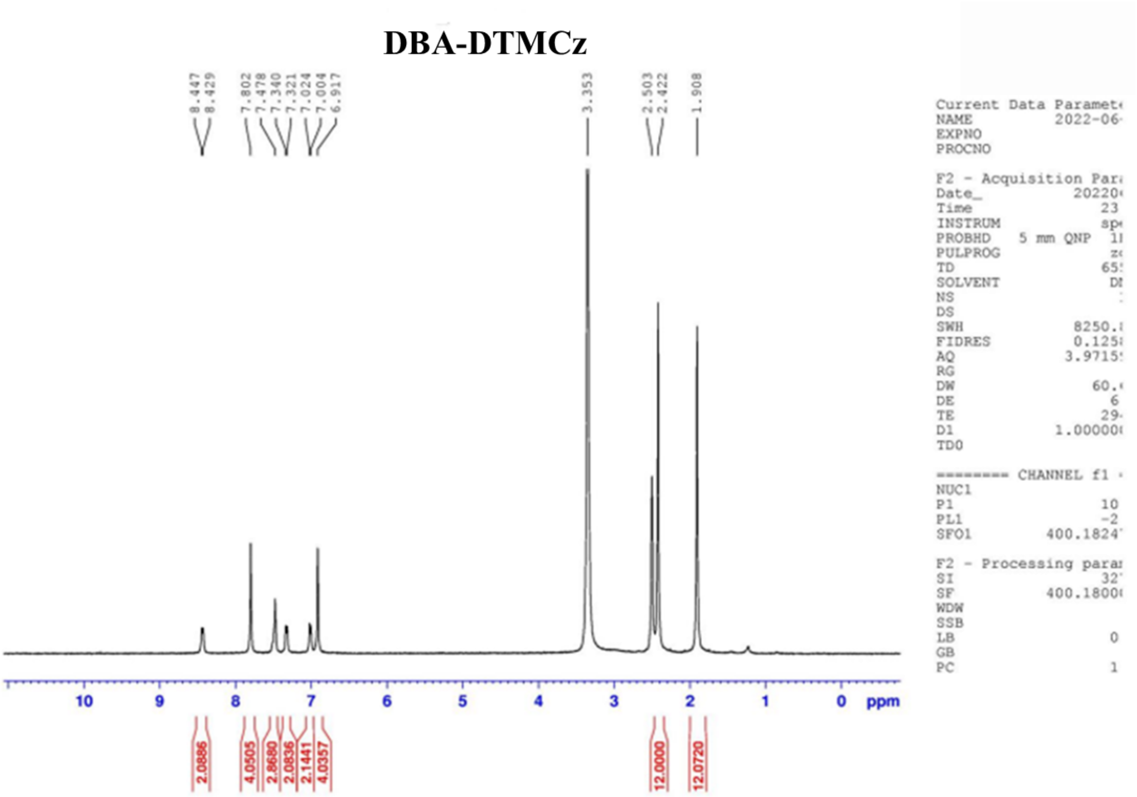

Supplementary Fig. 21 Proton NMR of 3,11-bis(1,3,6,8-tetramethyl-9H-carbazol-9-yl)-5,9-dioxa-13b-boranaphtho[3,2,1-de]anthracene (DBA-DTMCz).

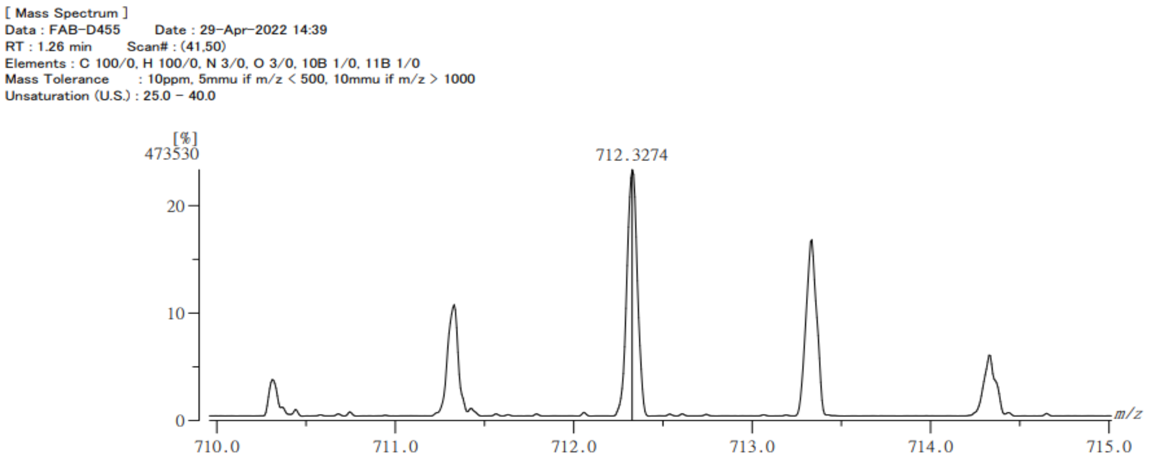

Supplementary Fig. 22 High resolution mass spectrometry of 3,11-bis(1,3,6,8-tetramethyl-9H-carbazol-9-yl)-5,9-dioxa-13b-boranaphtho[3,2,1-de]anthracene (DBA-DTMCz).

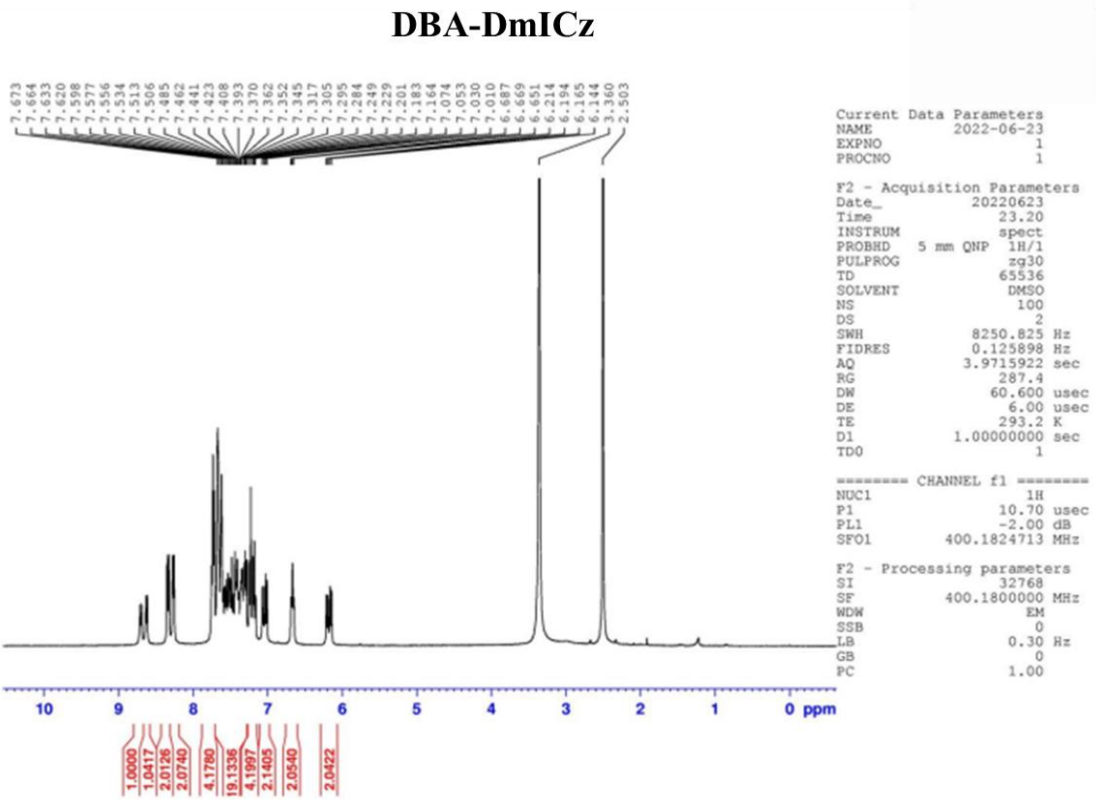

Supplementary Fig. 23 Proton NMR of 3,11-bis(5-phenylindolo[3,2-a]carbazol-12(5H)-yl)-5,9-dioxa-13b-boranaphtho[3,2,1-de]anthracene (DBA-DmICz).

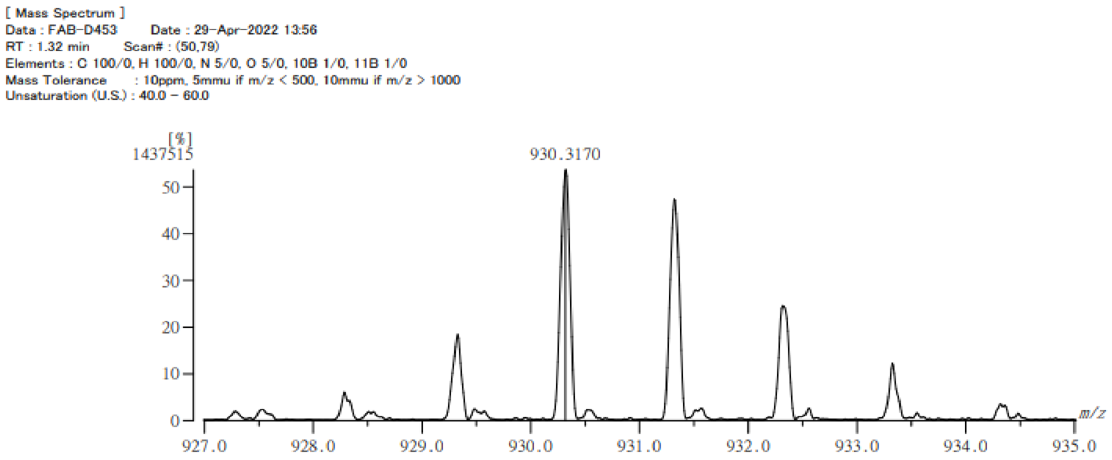

Supplementary Fig. 24 High resolution mass spectrometry of 3,11-bis(5-phenylindolo[3,2-a]carbazol-12(5H)-yl)-5,9-dioxa-13b-boranaphtho[3,2,1-de]anthracene (DBA-DmICz).

DBA-mICz

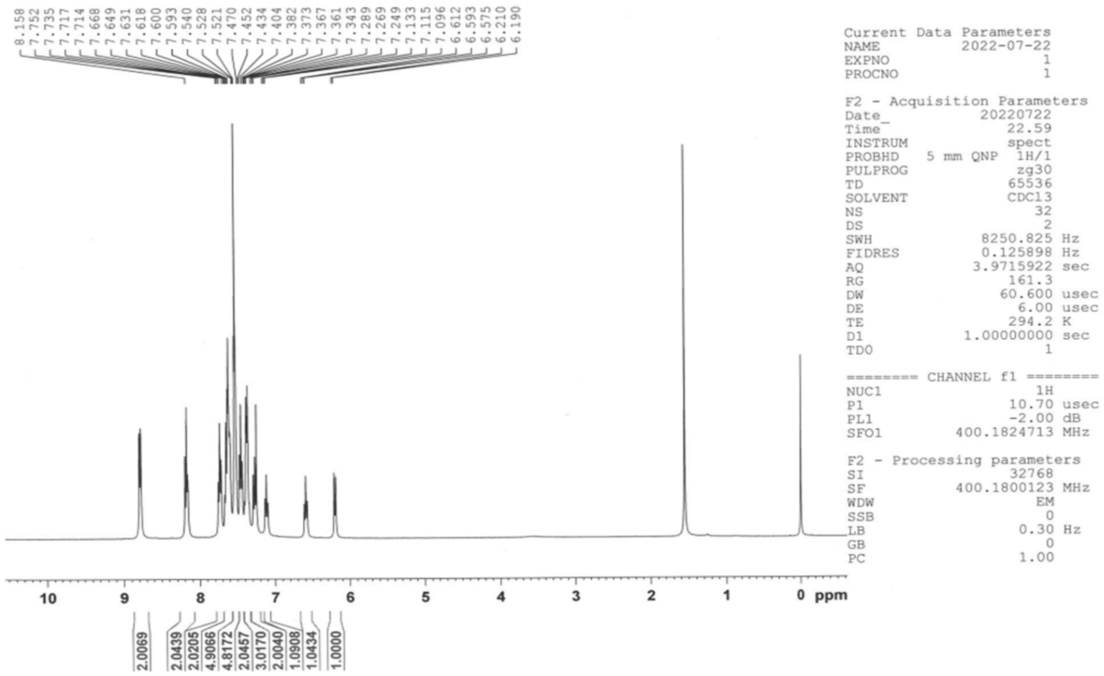

Supplementary Fig. 25 Proton NMR of 12-(5,9-dioxa-13b-boranaphtho[3,2,1-de]anthracen-7-yl)-5-phenyl-5,12-dihydroindolo[3,2-a]carbazole (DBA-mICz).

DBA-mICz

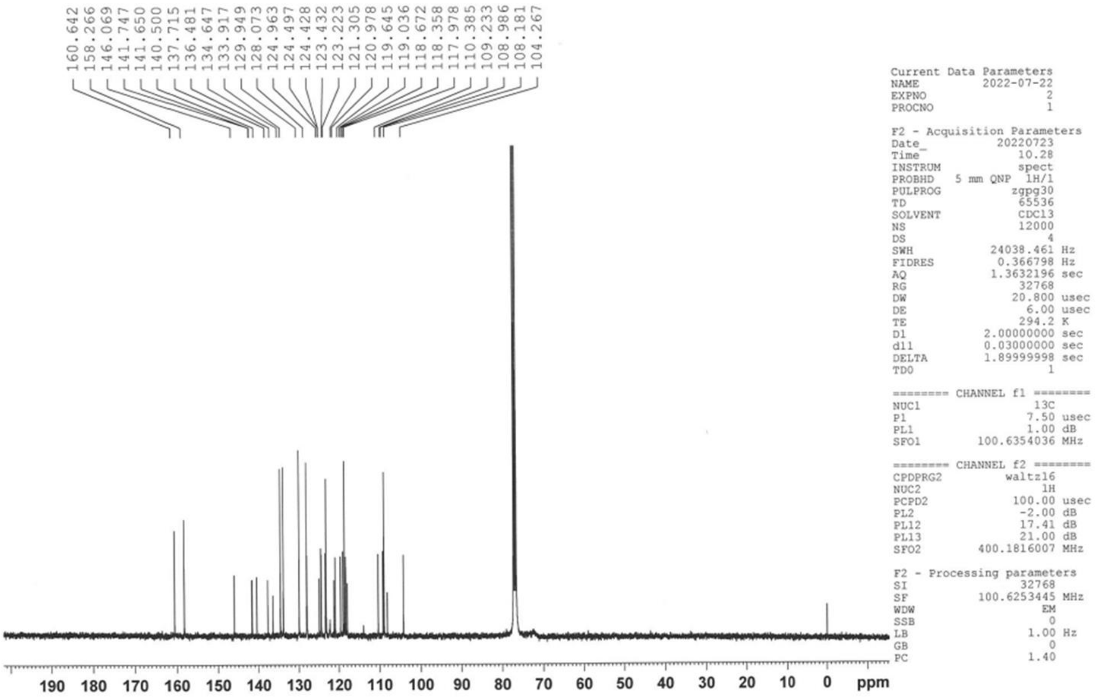

Supplementary Fig. 26 Carbon NMR of 12-(5,9-dioxa-13b-boranaphtho[3,2,1-de]anthracen-7-yl)-5-phenyl-5,12-dihydroindolo[3,2-a]carbazole (DBA-mICz).

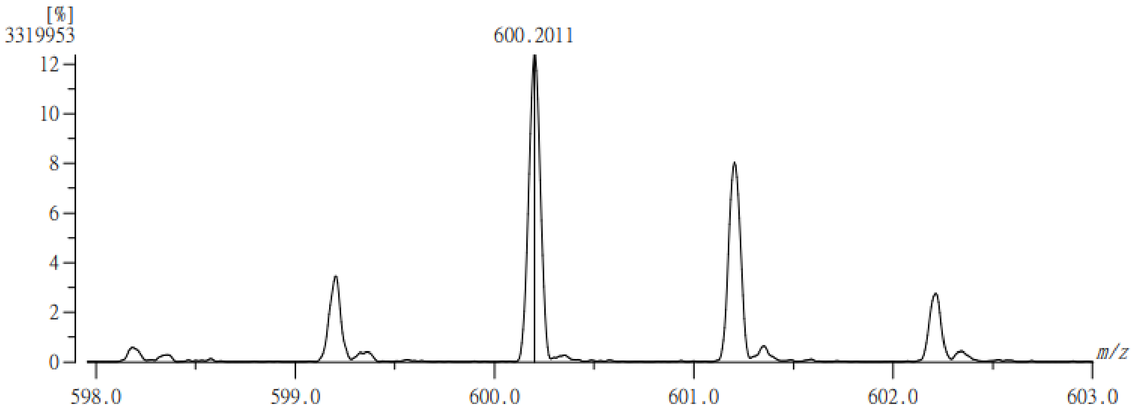

Supplementary Fig. 27 High resolution mass spectrometry of 12-(5,9-dioxa-13b-boranaphtho[3,2,1-de]anthracen-7-yl)-5-phenyl-5,12-dihydroindolo[3,2-a]carbazole (DBA-mICz).

Supplementary Tables

Supplementary Table 1 Calculated rate constant of 30% of DBA-DmICz and DBA-DTMCz in DBFPO host film.

	Φ_{PL}	Φ_p	Φ_d	Φ_{RISC}	τ_p (ns)	τ_d (μs)	k_r^S (10^7 /s)	k_{ISC} (10^7 /s)	k_{RISC} (10^6 /s)	k_{nr}^T (10^4/s)
DBA-DmICz	0.95	0.70	0.25	0.84	37.3	1.94	1.87	0.81	0.62	8.04
DBA-DTMCz	0.99	0.51	0.48	0.98	36.0	0.92	1.42	1.37	2.10	2.22

Supplementary Table 2 The photophysical and electrochemical properties of DBA-mICz.

	λ_{abs} (nm)[a]	λ_{RTPL} (nm)[b]	FWHM (nm) [b]	S_1 (eV)[c]	T_1 (eV)[d]	ΔE_{ST} (eV) [e]	HOMO (eV)[f]	LUMO (eV)[g]	PLQY[h]
DBA-mICz	360, 375	442	54	3.14	2.96	0.18	5.81	2.75	0.83

(a) UV-Vis absorption wavelengths measured in 10^{-5} M of toluene solution. (b) The maximum photoluminescence emission measured in toluene at 300 K. (c) Calculated by the onset of PL spectrum in toluene at 300 K. (d) Calculated by the onset of phosphorescence spectrum in toluene at 77 K with 30 ms of delaying. (e) $\Delta E_{ST} = S_1$-T_1. (f) HOMO level energy measured by cyclic voltammetry (CV) method. (g) LUMO=HOMO-optical band gap. (h) The absolute PLQY value measured in 30% doped DBFPO film.

Supplementary Table 3 Calculated rate constant of 30% of DBA-mICz in DBFPO host film.

	Φ_{PL}	Φ_p	Φ_d	Φ_{RISC}	τ_p (ns)	τ_d (μs)	k_r^S (10^7 /s)	k_{ISC} (10^7 /s)	k_{RISC} (10^6 /s)	k_{nr}^T (10^4/s)
DBA-mICz	0.85	0.64	0.21	0.59	45.90	2.04	1.40	0.76	0.45	20.2

Supplementary Table 4 TADF Device performances of DBA-DmICz and DBA-DTMCz with 20% of doping concentration in DBFPO host device.

	Turn on voltage[a] (V)	Driving voltage[b] (V)	Current Efficiency (Cd/A) (Max / 1,000 cd/m^2)	EQE (%) (Max / 1,000 cd/m^2)	Max emission peak (nm)[c]	FWHM (nm) [c]	CIE coordinates (1,000 cd/m^2)
20% DBA-DmICz	3.2	5.0	52.7 / 45.8	32.2 / 28.6	479	66	(0.15, 0.28)
20% DBA-DTMCz	3.2	5.0	56.4 / 52.6	36.3 / 33.3	476	58	(0.14, 0.25)

(a)Turn on voltage at 1 cd/m^2, (b) Driving voltage at 1,000 cd/m^2, (c) Measured at 1,000 cd/m^2.

Supplementary Table 5 TADF Device performances of DBA-mICz with 30 and 40% of doping concentration in DBFPO host device.

	Turn on voltage[(a)] (V)	Driving voltage[(b)] (V)	Current Efficiency (Cd/A) (Max / 1,000 cd/m²)	EQE (%) (Max / 1,000 cd/m²)	Max emission peak (nm)[(c)]	FWHM (nm)[(c)]	CIE coordinates (1,000 cd/m²)
30% DBA-mICz	3.2	5.3	23.9 / 17.6	18.6 / 14.6	467	67	(0.15, 0.18)
40% DBA-mICz	3.2	5.2	23.8 / 19.5	17.3 / 14.9	471	68	(0.15, 0.21)

(a)Turn on voltage at 1 cd/m², (b) Driving voltage at 1,000 cd/m², (c) Measured at 1,000 cd/m².

Supplementary Table 6 TRPL decay lifetime and calculated rate constant of FRET and DET in DBA-DmICz and DBA-DTMCz HF system with 1% of *v*-DABNA.

	Dopant concentration (%)	τ_p (ns)	τ_d (μs)	k_{FRET} (10^7/s)	k_{DET} (10^5/s)
**DBA-DmICz**	0	37.30	1.94		
	1	15.40	1.22	3.81	1.94
**DBA-DTMCz**	0	36.00	0.92		
	1	14.40	0.53	4.26	1.70

Supplementary Table 7 TRPL decay lifetime and calculated rate constant of FRET and DET in 20% of DBA-DTMCz and pMDBA-DI HF HF system with 1% of TBPe.

	Dopant concentration (%)	τ_p (ns)	τ_d (μs)	k_{FRET} (10^7/s)	k_{DET} (10^5/s)
**DBA-DTMCz**	0	36.10	0.96		
	1	14.90	0.58	3.38	2.48
***p*MDBA-DI**	0	30.00	1.96		
	1	15.90	0.89	2.97	3.12

The rate constant of FRET and DET can be calculated by: [3]

The decay rates of singlet (S_1) and triplet (T_1) excitons densities can be described by equation (1) and (2).

$$\frac{dS_1}{dt} = -S_1\left(k_{r,s} + k_{nr,s} + k_{ISC} + k_{FRET}\right) + T_1 k_{RISC} \quad (1)$$

$$\frac{dT_1}{dt} = -T_1\left(k_{r,T} + k_{nr,T} + k_{RISC} + k_{DET}\right) + S_1 k_{ISC} \quad (2)$$

Equation (1) and (2) can be solved by following biexponential decay

$$S_1, T_1 = A_1\exp(-k_{PF}t) + A_2\exp(-k_{DF}t) \qquad (3)$$

Where A_1 and A_2 are the prompt intensity and delayed intensity, respectively.

The decay rates for prompt and delayed fluorescence can be expressed as:

$$(k_{r,s} + k_{nr,s} + k_{ISC} + k_{FRET} - k_{PF})(k_{r,T} + k_{nr,T} + k_{RISC} + k_{DET} - k_{PF}) - k_{ISC}k_{RISC} = 0 \qquad (4)$$

$$(k_{r,s} + k_{nr,s} + k_{ISC} + k_{FRET} - k_{DF})(k_{r,T} + k_{nr,T} + k_{RISC} + k_{DET} - k_{DF}) - k_{ISC}k_{RISC} = 0 \qquad (5)$$

Assuming of,

i) $k_{PF} \gg k_{DF}$

ii) $k_{r,S}$, k_{ISC}, $k_{FRET} \gg k_{nr,S}$, $k_{nr,T}$, $k_{r,T}$ k_{DET}

Equation S4 and S5 can give:

$$k_{FRET} \approx k_{PF} - k_{r,S} - k_{ISC} \qquad (6)$$

$$k_{DET} \approx \frac{k_{PF}k_{DF} - k_{RISC}(k_{r,s} + k_{FRET})}{k_{r,s} + k_{ISC} + k_{FRET}} - k_{nr,T} \approx k_{DF} - k_{nr,T} - k_{RISC} + \frac{k_{RISC}k_{ISC}}{k_{PF}} \qquad (7)$$

Supplementary Table 8 Photophysical properties and rate constants of TADF materials in DBFPO host film at various doping concentration of 10-100%.

	Doping conc. (%)	τ_p (ns)	τ_d (μs)	Φ_{PL}	Φ_p	Φ_d	k_{PF} (10^7 /s)	k_{DF} (10^5 /s)	k_{ISC} (10^6 /s)	k_{RISC} (10^6 /s)	k_{CQ} (10^5/s)
DBA-mICz	10	45.9	2.46	0.90	0.64	0.26	2.18	4.07	7.82	-	1.19
	30	45.9	2.04	0.85	0.64	0.21	2.18	4.90	7.60	0.45	2.09
	60	45.9	1.28	0.75	0.63	0.12	2.18	7.81	7.99	-	5.42
	100	43.9	0.91	0.70	0.61	0.09	2.28	10.9	8.72	-	8.47
DBA-DmICz	10	37.3	2.12	0.97	0.71	0.26	2.68	4.72	8.04	-	0.67
	30	37.3	1.94	0.95	0.70	0.25	2.68	5.16	0.81	0.62	0.85
	60	37.3	1.59	0.92	0.69	0.23	2.68	6.29	8.17	-	1.71
	100	37.0	1.37	0.83	0.69	0.14	2.70	7.30	8.41	-	4.15
DBA-DTMCz	10	36.0	0.97	1.00	0.51	0.49	2.78	10.24	13.72	-	0.87
	30	36.0	0.92	0.99	0.51	0.48	2.78	10.91	13.70	2.10	1.12
	60	36.0	0.84	0.97	0.51	0.46	2.78	11.90	13.60	-	1.68
	100	35.2	0.80	0.86	0.50	0.36	2.84	12.52	14.18	-	4.25

Supplementary Methods

General information and characterization

All reagents were purchased from commercial suppliers, Sigma-Aldrich, TCI (SEJINCI) and SK chemicals, and they were used without further purification otherwise stated. All solvents were used without additional purification. Silica filtration used silica with a mesh size of 200-300. All reaction progress were monitored by using thin layer chromatography (TLC). 1,4,5,8,9,11-exaazatriphenylene-hexacarbonitrile (HATCN) was purchased from OSM. 1,1-Bis[(di-4-tolylamino)phenyl]cyclo-hexane (TAPC) and 2,2′,2″-(1,3,5-benzinetriyl)-tris(1-phenyl-1-H-benzimidazole) (TPBi), were purchased from Jilin OLED Material Tech Co., Ltd. 3,5-di(9Hcarbazol-9-yl)-N,N-diphenylaniline(DCDPA)[1] and 2,8-bis(diphenylphosphineoxide) dibenzofuran(DBFPO)[2] were synthesized based on the previously reported methods. For the characterization of the photophysical properties, all materials were prepared in toluene solution at a concentration of 1×10^{-5} M. The UV-vis absorption spectrum was measured by a V-750 spectrophotometer (JASCO). The solution PL spectrum and low temperature (77 K) phosphorecence spectrum were measured by a FP-8500 spectrofluorometer (JASCO). The absolute PLQY values in doped films were measured by connecting an integrating sphere to the same spectrofluorometer. These doped films were also used for the measuring transient PL measurements. Transient PL was measured when photon counts were reached until 10,000 in a nitrogen environment using a Quantaurus-Taufluorescence lifetime measurement system (C11367-03, Hamamatsu Photonics Co.). Their thermal properties were investigated by differential scanning calorimetry (DSC) and thermogravimetric analysis (TGA). The glass transition temperatures (T_g) were measured by the DSC graph, and the decomposition temperatures (T_d) were measured by TGA at 5% weight loss. Electrochemical analysis was performed using an EC epsilon electrochemical analysis equipment. To measure the cyclic voltammetry (CV) characteristics, platinum, material coated 150 nm of ITO glass, and Ag wire in 0.01 M $AgNO_3$, 0.1 M tetrabutyl ammonium perchlorate (Bu_4NClO_4), and acetonitrile solution were used as counter, working, and reference electrodes, respectively. For supporting electrolyte, 0.1 M tetrabutyl ammonium perchlorate in acetonitrile solution was used. Using an internal ferrocene/ferrocenium (Fc/Fc^+) standard, the potential values were converted to the saturated calomel electrode scale. To verify molecular structures of the synthesized materials, ^1H and ^{13}C NMR spectrum was measured using Bruker Avance 400 NMR spectrometer. The proton NMR signals were denoted as *s* (singlet), *d* (doublet), *t* (triplet), *dd* (doublets of doublet). High-resolution mass spectra were performed using JEOL JMS-600W Gas Chromatography

Mass spectrometer. ^{13}C NMRs of DBA-DmICz and DBA-DTMCz were not able to record due to their poor solubility in common NMR solvents.

Device fabrication

Prior to OLED device fabrication, indium-tin-oxide (ITO)-coated glass substrates (50 nm, sheet resistance of 10 Ω/m^2) were cleaned in an ultrasonic bath with isopropyl alcohol and acetone sequentially for 10 min each and then rinsed with deionized water. The glass substrates were dried using nitrogen and treated in UV-ozone chamber for 10 min. All organic layers and the metal cathode were deposited on the cleaned ITO glass by a vacuum evaporation technique under a vacuum pressure of $\sim 1 \times 10^{-7}$ Torr. The deposition rate of all organic layers was around 0.5 Å/s. The deposition rates of lithium fluoride (LiF) and aluminum (Al) were maintained at 0.1, 4.0 Å/s, respectively. After deposition process, the devices were encapsulated with a glass cover and an UV-curable resin inside a nitrogen-filled glovebox. The emitting areas were 4 mm^2 for all the samples in this work. *J-V* and *L-V* characteristics of the fabricated OLED devices were measured by using Keithley 2635A SMU and Konica Minolta CS-100A, respectively. EL spectra and CIE 1931 color coordinates were obtained using a Konica Minolta CS-2000 spectroradiometer. All measurements were performed in ambient conditions.

Theoretical Calculation

All the molecular simulations were performed using the Schrödinger 2022-1 program. The optimized geometry of ground state, and HOMO, LUMO distribution were calculated using the "Optimization" tool at the B3LYP/6-31G level. The energy levels of HOMO, LUMO, S_1, and T_1 energies were calculated by "Optoelectronics Calculations" tool at the B3LYP/6-31G level. NTO calculation and transition dipole moments were performed by "Single Point Energy" tool at the PBE0/6-31G level. Spin-orbit coupling matrix element value was calculated by the "Single Point Energy" tool at the PBE0 / DYALL-2ZCVP_ZORA-J-PT-GEN level. The optical simulations of dipole orientation value were performed using SETFOS 4.3 (Fluxim) program. The refractive index value, extinction coefficient, photoluminescence spectrum of EML, and thickness of each layer values were measured by ellipsometry and utilized as input parameters.

Synthetic Procedures and Analysis

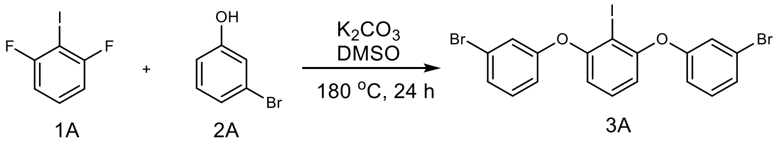

Supplementary Fig. 28 Synthesis of 3,3'-((2-iodo-1,3-phenylene)bis(oxy))bis bromobenzene) (3A).

1,3-difluoro-2-iodobenzene 1A (3.0 g, 12.5 mmol), 3-bromophenol 2A (5.19 g, 30.0 mmol), K_2CO_3 (6.91 g, 50 mmol) and 15 mL of DMSO were added into a two neck round bottom flask equipped with a condenser. The whole mixture was stirred at room temperature while argon purging for 15 minutes. Later, the reaction was held at 180°C for 24 hours in a vigorous stirring condition using a laboratory magnetic stir. After the reaction completion, the crude mixture was poured into ice water (200 mL) and stirred for 4 hours at room temperature. Then, the precipitate was collected and dissolved in ethylacetate completely. The obtained clear solution was subjected to pass-through a silica bed twice. Collected crude mixture was concentrated under a reduced pressure using a rotary evaporator, then, little amount of *n*-hexane was added and stirred at room temperature for an hour. The colorless precipitate was collected and washed several time with cold *n*-hexane and dried in an oven (65°C) for 6 hours. Yield: 90 %; colorless solid; ^{1}H NMR (400 MHz, $CDCl_3$) δ: 7.21-7.28 (m, 5H), 7.17 (s, 2H), 6.96 (d, *J*= 8.0 Hz, 2H), 6.71 (d, *J*= 8.0 Hz, 2H).

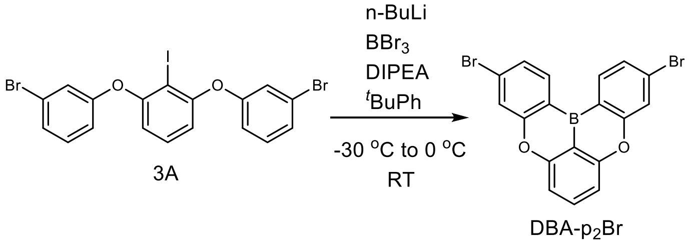

Supplementary Fig. 29 Synthesis of 3,11-dibromo-5,9-dioxa-13b-boranaphtho[3,2,1-de]anthracene (DBA-p2Br).

3,3'-((2-iodo-1,3-phenylene)bis(oxy))bis(bromobenzene) 3A (3 g, 5.49 mmol) was added into a two neck round bottom flask equipped with condenser and completely dissolved in 15 mL of *tert*-Butylbenzene at room temperature. Then, argon purging was held for 20 minutes while evacuation. The reaction mixture was brought to -30°C, and stirred at that temperature for 20 minutes. Then, *n*-Butyllithium solution (1.6 M, 3.6 mL) in hexane was added drop wisely over 10 minutes. And the reaction mixture kept stirring at above temperature for an hour. After that,

boron tribromide 99% (0.57 mL, 6.04 mmol) was added slowly over 5 minutes duration. The reaction mixture was kept stirring until it becomes room temperature. Once it reached room temperature, the reaction condition was brought to 0°C, and *N*,*N*-Diisopropylethylamine (4.79 mL, 27.47 mmol) was injected drop wisely and after the addition, reaction mixture stirred at room temperature for 15 minutes. Then, the reaction temperature was increased to 120°C and stirred at that temperature for 14 hours. After the reaction completion, the crude mixture was poured into excess amount of ethanol and stirred for 2 hours at room temperature. Precipitate was collected and washed with excess amount of ethanol and phosphate buffer solution. After drying under 80°C, the solid was completely dispersed in chloroform and filtered through a celite and silica pad, and then the clear solution was concentrated using a rotary evaporator. After complete evaporation, little amount of dichloromethane was added at room temperature and followed by cold methanol was added slowly until clear solution turns cloudy. After stirring at low temperature for 6 hours, formed colorless fluffy solid was collected and washed with methanol to obtain the target acceptor of DBA-p2Br. Yield: 72 %; colorless solid; ^{1}H NMR (400 MHz, DMSO-d_6) δ: 8.08 (d, *J*= 8.0 Hz, 2H), 7.40-7.43 (m, 3H), 7.36 (d, *J*= 7.6 Hz, 2H), 6.94 (d, *J*= 7.6 Hz, 2H).

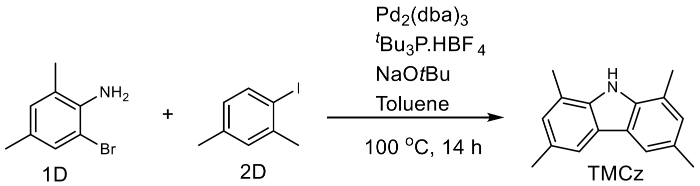

Supplementary Fig. 30 Synthesis of 1,3,6,8-tetramethyl-9H-carbazole (TMCz).

2-bromo-4,6-dimethylaniline 1D (4 g, 19.99 mmol), 1-iodo-2,4-dimethylbenzene 2D (4.87 g, 20.99 mmol), tris(dibenzylideneacetone)dipalladium(0) (1.09 g, 1.19 mmol), tri-*tert*-butylphosphonium tetrafluoroborate (0.69 g, 2.39 mmol) and sodium *tert*-butoxide (3.45 g, 35.98 mmol) was added into a two neck round bottom flask equipped with a condenser, and subjected to vacuum for 10 minutes. Anhydrous toluene of 30 mL was injected followed by argon purging. The mixture was stirred at 100°C for 4 hours and reaction progress was monitored using thin layer chromatography. Then, mixture of tris(dibenzylideneacetone) dipalladium(0) (0.545 g, 0.59 mmol) and tri-*tert*-butylphosphonium tetrafluoroborate (0.345 g, 1.19 mmol) in 5 mL toluene was added in to the above mixture and kept string at the same temperature for another 10 hours. After 14 hours of total reaction, the reaction mixture was filtered through a silica pad. Then, worked up using dichloromethane (60 mL) and water (25 mL) twice. The collected organic layer was dried over anhydrous sodium sulfate. After filtration,

crude mixture was evaporated under a reduced pressure. Finally, the crude mixture was dissolved in dichloromethane, and excess amount of *n*-hexane was added slowly and stirred for 8 hours at room temperature. And off-white precipitate was collected and dried under a vacuum to obtain the donor intermediate of TMCz. Yield: 58 %; off-white solid; ^{1}H NMR (400 MHz, $CDCl_3$) δ: 7.66 (s, 2H), 7.57 (s, 1H, amine), 7.02 (s, 2H), 2.5 (s, 12H, methyl).

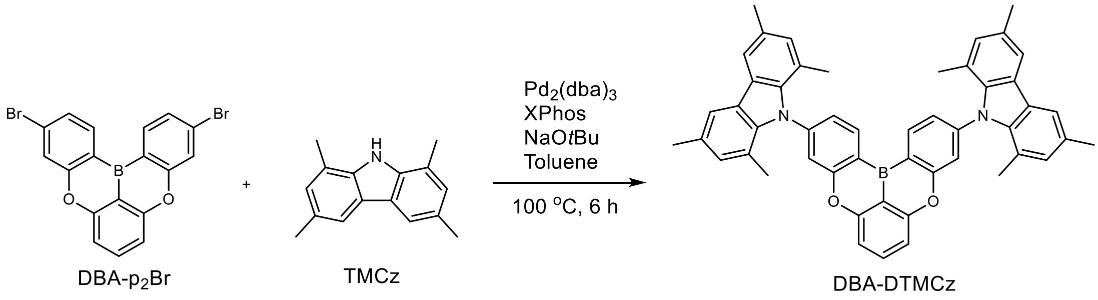

Supplementary Fig. 31 Synthesis of 3,11-bis(1,3,6,8-tetramethyl-9H-carbazol-9-yl)-5,9-dioxa-13b-boranaphtho[3,2,1-de]anthracene (DBA-DTMCz).

3,11-dibromo-5,9-dioxa-13b-boranaphtho[3,2,1-de]anthracene DBA-p2Br (1.0 g, 2.33 mmol), 1,3,6,8-tetramethyl-9H-carbazole TMCz (1.09 g, 4.88 mmol), tris(dibenzylideneacetone)dipalladium(0) (0.085 g, 0.09 mmol), 2-dicyclohexylphosphino-2′,4′,6′-triisopropylbiphenyl (0.089 g, 0.18 mmol), sodium *tert*-butoxide (0.62 g, 6.54 mmol) and anhydrous toluene (18 mL) were added in to a dried two neck round bottom flask equipped with a condenser at room temperature. Then, the above mixture kept stirring at 100℃ for 6 hours under an inert condition. After complete disappearing of stating materials, crude mixture was worked up using chloroform (30 mL*2) and water (75 mL). Collected organic layer was dried over anhydrous magnesium sulfate and filtered using a filter paper. Then, collected solution was filtered through a silica/ celite pad twice. Finally, the filtered solution was concentrated under a reduced pressure, when 95% of solvent got evaporated, *n*-hexane was added slowly into the flask. Further, it was kept stirring at room temperature for 2.5 hours and the greenish-yellow solid was collected and washed with excess amount of *n*-hexane to obtain the target emitter of 3,11-bis(1,3,6,8-tetramethyl-9H-carbazol-9-yl)-5,9-dioxa-13b-boranaphtho[3,2,1-de]anthracene DBA-DTMCz. Yield: 81%; greenish-yellow solid; ^{1}H NMR (400 MHz, DMSO-d_6) δ: 8.43 (d, *J*= 7.2 Hz, 2H), 7.80 (s, 4H), 7.47 (m, 3H), 7.33 (d, *J*= 7.6 Hz, 2H), 7.01 (d, *J*= 8.0 Hz, 2H), 6.91 (s, 4H), 2.42 (s, 12H, methyl), 1.90 (s, 12H, methyl); HRMS (ESI) *m*/*z*: Anal. calcd. For $C_{50}H_{41}BN_2O_2$, 712.3261; **found, 712.3274**.

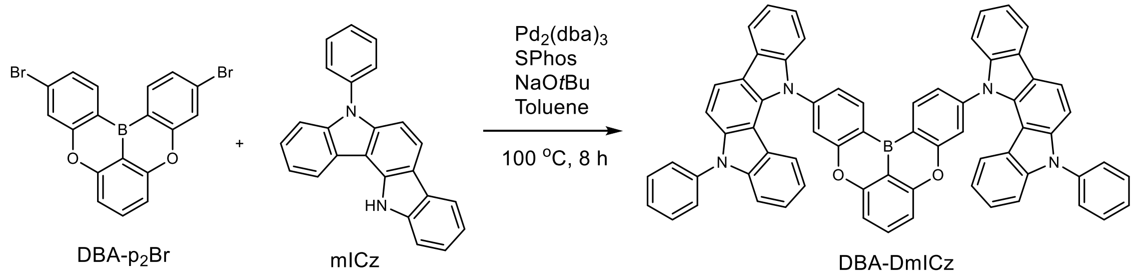

Supplementary Fig. 32 Synthesis of 3,11-bis(5-phenylindolo[3,2-a]carbazol-12(5H)-yl)-5,9-dioxa-13b-boranaphtho[3,2,1-de]anthracene (DBA-DmICz).

3,11-dibromo-5,9-dioxa-13b-boranaphtho[3,2,1-de]anthracene DBA-p2Br (1.0 g, 2.33 mmol), 5-phenyl-5,12-dihydroindolo[3,2-a]carbazole mICz (1.63 g, 4.90 mmol), tris(dibenzylideneacetone)dipalladium(0) (0.085 g, 0.09 mmol), 2-dicyclohexylphosphino-2′,6′-dimethoxybiphenyl (0.076 g, 0.18 mmol), sodium *tert*-butoxide (0.62 g, 6.54 mmol) and anhydrous toluene (22 mL) were added in to a dried two neck round bottom flask equipped with a condenser at room temperature. Then, the above mixture kept stirring at 100°C for 8 hours under an inert condition. After complete disappearing of stating materials, crude mixture was worked up using chloroform (30 mL*3) and water (90 mL). Collected organic layer was dried over anhydrous magnesium sulfate and filtered using a filter paper. Then, collected solution was filtered through a silica/ celite pad twice. Finally, the filtered solution was concentrated under a reduced pressure, when 95% of solvent got evaporated, *n*-hexane was added slowly into the flask. Further, it was kept stirring at room temperature for 4 hours and the bright greenish solid was collected and dissolved completely in hot toluene, and *n*-hexane was added drop wisely until solid formation and kept stirring at room temperature for an hour, then precipitate was filtered and washed with excess amount of *n*-hexane to obtain the target emitter of 3,11-bis(5-phenylindolo[3,2-a]carbazol-12(5H)-yl)-5,9-dioxa-13b-boranaphtho[3,2,1-de] anthracene DBA-2MICz. Yield: 74 %; bright greenish solid; ^{1}H NMR (400 MHz, DMSO-d_6) δ: 8.70 (d, *J*= 8.0 Hz, 1H), 8.62 (d, *J*= 8.0 Hz, 1H), 8.30-8.35 (dd, *J*= 8.4, 2.4 Hz, 2H), 8.26 (d, *J*= 7.2 Hz, 2H), 7.71-7.75 (m, 4H), 7.29-7.67 (m, 19H), 7.16-7.28 (m, 4H), 7.01-7.07 (q, *J*= 7.2, 8.0 Hz, 2H), 6.67 (d, *J*= 7.2 Hz, 2H), 6.20 (d, *J*= 8.0 Hz, 1H), 6.15 (d, *J*= 8.4 Hz, 1H); HRMS (ESI) *m*/*z*: Anal. calcd. For $C_{66}H_{39}BN_4O_2$, 930.3166; **found, 930.3170**.

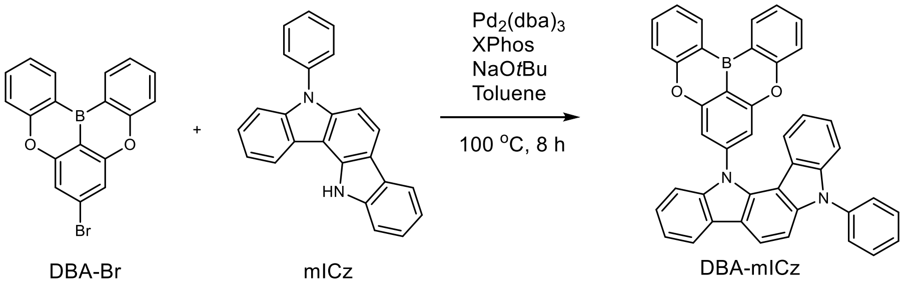

Supplementary Fig. 33 Synthesis of 12-(5,9-dioxa-13b-boranaphtho[3,2,1-de]anthracen-7-yl)-5-phenyl-5,12-dihydroindolo[3,2-a]carbazole (DBA-mICz).

7-bromo-5,9-dioxa-13b-boranaphtho[3,2,1-de]anthracene DBA-Br (2 g, 5.73 mmol), 5-phenyl-5,12-dihydroindolo[3,2-a]carbazole mICz (2.09 g, 6.30 mmol), tris(dibenzylideneacetone)dipalladium(0) (0.052 g, 0.06 mmol), 2-dicyclohexylphosphino-2′,4′,6′-triisopropylbiphenyl (0.054 g, 0.12 mmol), sodium *tert*-butoxide (1.54 g, 16.04 mmol) and anhydrous toluene (20 mL) were added in to a dried two neck round bottom flask equipped with a condenser at room temperature. Then, the above mixture kept stirring at 100°C for 10 hours under an inert condition. Crude mixture was worked up using dichloromethane and water. Collected organic layer was dried over anhydrous magnesium sulfate, and concentrated after the filtration. Finally, the crude mixture was purified using toluene and *n* hexane. Yield: 60 %; ^{1}H NMR (400 MHz, $CDCl_3$) δ: 8.78 (d, *J*= 6.4 Hz, 2H), 8.15-8.19 (m, 2H), 7.71-7.75 (m, 2H), 7.59-7.66 (m, 5H), 7.45 (t, *J*= 7.2 Hz, 2H), 7.34-7.48 (m, 3H), 7.24-7.28 (m, 1H), 7.11 (t, *J*= 7.6 Hz, 1H), 6.59 (t, *J*= 7.6 Hz, 1H), 6.20 (d, *J*= 8.0 Hz, 1H); ^{13}C NMR (100 MHz, $CDCl_3$) δ 160.64, 158.27, 146.07, 141.75, 141.65, 140.50, 137.72, 136.48, 134.65, 133.92, 129.95, 128.07, 124.96, 124.49, 124.43, 123.43, 123.22, 121.31, 120.98, 119.65, 119.04, 118.67, 118.36, 117.98, 110.39, 109.23, 108.99, 108.18, 104.27. HRMS (ESI) *m*/*z*: Anal. calcd. For $C_{42}H_{25}BN_2O_2$, 600.2009; **found, 600.2011**.

Supplementary References

1. Cho, Y.J & Lee, J.Y. Low Driving Voltage, High Quantum Efficiency, High Power Efficiency, and Little Efficiency Roll-Off in Red, Green, and Deep-Blue Phosphorescent Organic Light-Emitting Diodes Using a High-Triplet-Energy Hole Transport Material. *Adv. Mater*. **23**, 4568-4572 (2011).

2. Vecchi, P. A., Padmaperuma, A. B., Qiao, H., Sapochak, L. S. & Burrows, P. E. A dibenzofuran-based host material for blue electrophosphorescence. *Org. Lett*. **8**, 4211-4214 (2006).
3. Aizawa, N., Shikita, S. & Yasuda, T. Spin-Dependent Exciton Funneling to a Dendritic Fluorophore Mediated by a Thermally Activated Delayed Fluorescence Material as an Exciton-Harvesting Host. *Chem. Mater*. **29**, 7014-7022 (2017).
